# The Emerging Roles of RNA Modifications in Glioblastoma

**DOI:** 10.3390/cancers12030736

**Published:** 2020-03-20

**Authors:** Zhen Dong, Hongjuan Cui

**Affiliations:** 1 State Key Laboratory of Silkworm Genome Biology, Institute of Sericulture and Systems Biology, College of Biotechnology, Southwest University, Beibei, Chongqing 400716, China; 2 Cancer Center, Medical Research Institute, Southwest University, Beibei, Chongqing 400716, China; 3 Engineering Research Center for Cancer Biomedical and Translational Medicine, Southwest University, Beibei, Chongqing 400716, China; 4 Chongqing Engineering and Technology Research Center for Silk Biomaterials and Regenerative Medicine, Southwest University, Beibei, Chongqing 400716, China

**Keywords:** m^6^A, RNA modification, glioblastoma, epi-transcriptome, RNA processing

## Abstract

Glioblastoma (GBM) is a grade IV glioma that is the most malignant brain tumor type. Currently, there are no effective and sufficient therapeutic strategies for its treatment because its pathological mechanism is not fully characterized. With the fast development of the Next Generation Sequencing (NGS) technology, more than 170 kinds of covalent ribonucleic acid (RNA) modifications are found to be extensively present in almost all living organisms and all kinds of RNAs, including ribosomal RNAs (rRNAs), transfer RNAs (tRNAs) and messenger RNAs (mRNAs). RNA modifications are also emerging as important modulators in the regulation of biological processes and pathological progression, and study of the epi-transcriptome has been a new area for researchers to explore their connections with the initiation and progression of cancers. Recently, RNA modifications, especially m^6^A, and their RNA-modifying proteins (RMPs) such as methyltransferase like 3 (METTL3) and α-ketoglutarate-dependent dioxygenase alkB homolog 5 (ALKBH5), have also emerged as important epigenetic mechanisms for the aggressiveness and malignancy of GBM, especially the pluripotency of glioma stem-like cells (GSCs). Although the current study is just the tip of an iceberg, these new evidences will provide new insights for possible GBM treatments. In this review, we summarize the recent studies about RNA modifications, such as N^6^-methyladenosine (m^6^A), N^6^,2′O-dimethyladenosine (m^6^A_m_), 5-methylcytosine (m^5^C), N^1^-methyladenosine (m^1^A), inosine (I) and pseudouridine (ψ) as well as the corresponding RMPs including the writers, erasers and readers that participate in the tumorigenesis and development of GBM, so as to provide some clues for GBM treatment.

## 1. Introduction

Glioblastoma (GBM), a World Health Organization (WHO) grade IV glioma, is the most prevalent, malignant and lethal intrinsic tumor in the central nervous system [1]. It accounts for about 50% of all brain neoplasms and has a median survival time of approximately 14.6 months [2]. The annual worldwide occurrence of this disease is about five cases per 100,000 people [3]. Currently, the origin of GBM is mainly the poorly differentiated glial cell, which is characterized with nuclear atypia, cellular polymorphism and high mitotic activity [4]. Since GBM is very aggressive and invasive, it is difficult to remove all the neoplastic lesions from the brain by using surgical resection. Subsequently, the patients typically receive 30-week radiotherapy and an adjuvant chemotherapeutic alkylating drug, temozolomide (TMZ) [3]. However, since GBM is highly heterogeneous, the effect is limited and no remarkable progression has been achieved yet. Therefore, it is urgent to develop new therapeutic strategies to treat GBM; however, the natural characteristics of GBM remain not fully understood.

To know how GBM initiates and develops, the molecular characteristics of GBM should be accurately determined. Actually, many genetic, epigenetic, metabolic and immunologic profiles in GBM have been described in recent years [5,6,7,8,9]. These landscapes of GBM have greatly expanded our knowledge about GBM, thereby providing clues for the treatment of this disease. Particularly, covalent modifications in DNA and histones, also known as that main concept of epigenetics, play important roles in regulating cell fate determination, cell proliferation, cellular metabolism and even many pathological processes [10,11,12,13,14]. Recently, the RNA world has become a new area to be explored for cancer therapy. Both coding and non-coding mRNAs have many diverse functions in addition to their traditional functions. Studies on RNA processing, editing, splicing, polyadenylation, post-transcription of RNA and RNA-binding proteins are developing very fast [15,16,17,18], leading to a new area called the epi-transcriptome. Covalent modifications in RNA and their connections with cancers are also an area of concentrated activity in cancer biology research [19].

Recently, RNA modifications, as known as RNA epigenetics, were found to be present in almost all the cellular RNAs in archaeobacteria, bacteria, prototists, plants, fungi, animals and viruses. Even in mitochondria, there are also some chemical modifications in mitochondrial DNA-encoded RNAs [20]. More than 170 kinds of covalent modifications have been found in RNAs at present [21,22], however, the functional implications of most of them remain unravelled. These kinds of biological modifications exercise important regulation functions during both normal and pathological bioprocesses and tissue development. Importantly, RNA modifications, especially N^6^-methyladenosine (m^6^A) modification, are shown to be essential for tumor development [22,23,24,25].

In this review, we summarize the most recent advances in RNA modifications, especially the functions of m^6^A writers, erasers and readers, and their molecular mechanisms of action in GBM, so as to provide some clues for the development of new strategies for GBM treatment.

## 2. RNA Modifications

RNA modifications refer to dynamic, reversible covalent alterations present in the oligonucleotides of RNAs, including mRNAs, tRNAs, rRNAs and spliceosomal RNAs (sRNAs). These changes are commonly present in the transcriptome. Since 1957, when pseudouridine (ψ, also known as the fifth RNA nucleotide) was discovered by Davis and Allen [26], at least 170 kinds of RNA modifications have been identified [26]. Actually, m^7^G (7-methylguanosine, also known as 5′ cap) and 3′ poly(A) tail are well-known modifications in mRNA [27]. In 2012, the NGS method was developed, and since then, many widespread and conserved internal RNA modifications in RNAs were identified [22].

In eukaryotic RNA, the most commonly found modifications are N^6^-methyladenosine (m^6^A), N^6^, 2′O-dimethyladenosine (m^6^A_m_), 5-methylcytosine (m^5^C), 5-hydroxymethylcytosine (hm^5^C), N^1^-methyladenosine (m^1^A), inosine (I), N^4^-acetylcytidine (ac4C), ψ, uridylation (U-tail), 2-methylthio-N^6^-(*cis*-hydroxyisopentenyl)adenosine (ms^2^i^6^A), 3-methylcytosine (m^3^C), m^7^G, 7-methylguanosine cap (m^7^Gpp(pN)), queuosine (Q), wybutosine and derivatives (yW), 5-methyluridine (m^5^U), 5-carbamoylmethyluridine (ncm^5^U), 5-methoxycarbonylmethyluridine (mcm^5^U), 5-methoxy-carbonylmethyl-2-thiouridine (mcm^5^s^2^U), dihydrouridine (D), 2′-O-methylnucleotide (Nm), N^6^-isopentenyladenosine (i^6^A), and 5′-phosphate monomethylation (m(pN)) [28,29,30]. Most of these modifications have been found in mRNAs. Moreover, eukaryotic tRNAs also contain many modifications (13 modifications per molecule on average) to maintain their chemical diversity, thereby contributing to decoding fidelity, folding efficiency, cellular stability and localization [31]. rRNAs also contain abundant modifications (>200 sites per molecule) including 2′-O-methyls, pseudouridines, and base methylations in the peptidyl transferase center, the A, P and E sites of tRNA- and mRNA binding [32], thereby maintaining their functions. In addition, sRNAs also contain moderate amounts of modifications (>50 sites per molecule), some of which play important roles in the RNA splicing actions [33].

**Figure 1:**
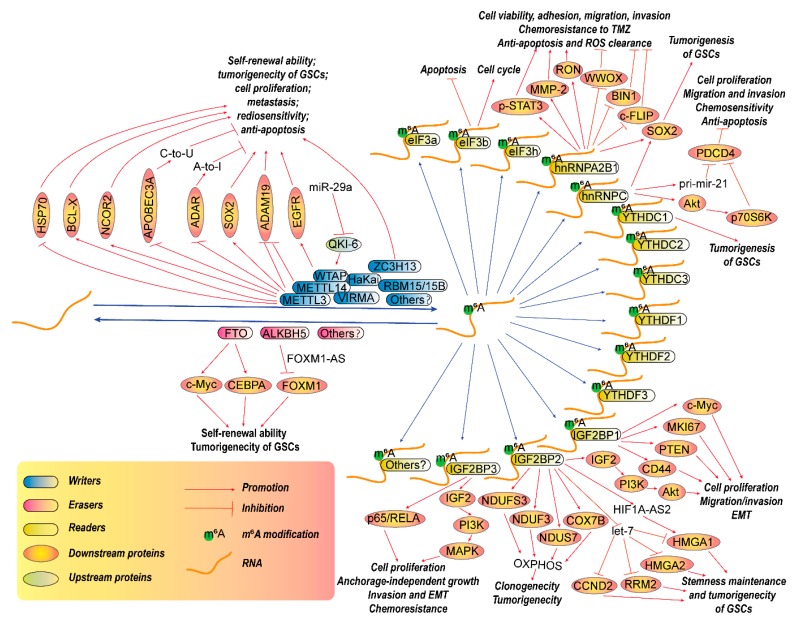
Functions of m6A modification and its writers, erasers and readers in GBM. Abbreviations: RNA-modifying proteins (RMPs): ALKBH5, α-Ketoglutarate-dependent dioxygenase alkB homolog 5; eIF3a/b/h, eukaryotic translation initiation factor 3a/b/h; FTO, Fat mass and obesity-associated protein; Hakai, Cbl proto-oncogene like 1, CBLL1; hnRNPA2B1, heterogeneous nuclear ribonucleoprotein A2/B1; hnRNPC, heterogeneous nuclear ribonucleoprotein C1/C2; IGF2BP1/2/3, insulin-like growth factor 2 mRNA-binding protein 1/2/3; METTLE3/14, methyltransferase like 3/14; RBM15/15B, RNA-binding motif protein 15/15B; VIRMA, Virilizer like m6A methyltransferase associated protein; WTAP, Wilms’ tumor 1-associated protein; YTHDF1/2/3, YTH N6-methyladenosine RNA binding protein 1/2/3; YTHDC1/2/3, YTH domain-containing 1/2/3; ZC3H13, zinc finger CCCH domain-containing protein 13. Others: A-to-I, adenosine-to-inosine; ADAM19, a disintegrin and metallopeptidase domain 19; ADAR1,adenosine deaminase RNA specific 1; APOBEC3A, apolipoprotein B mRNA editing enzyme catalytic subunit 3A; BCL-X, Bcl-2-like protein 1, BCL2L1; BIN1, bridging integrator 1; c-FLIP, cellular FLICE (FADD-like IL-1β-converting enzyme)-inhibitory protein; c-Myc, avian myelocytomatosis viral oncogene homolog; C-to-U, cytidine to uridine; CCND2, cyclin D2; CEBPA, CCAAT enhancer binding protein alpha; COX7B, mitochondrial cytochrome c oxidase subunit 7B; EGFR, epidermal growth factor receptor; EMT, epithelial-to-mesenchymal transition; FOXM1, forkhead box M1; FOXM1-AS, forkhead box M1-antisense RNA; GSCs, glioma stem-like cells; HIF1A-AS2, hypoxia-inducible factor 1 alpha-antisense RNA 2; HMGA1/2, high mobility group AT-hook 1/2; HSP70, heat shock protein 70; IGF2, insulin-like growth factor 2; m^6^A, N^6^-methyladenosine; MAPK, mitogen-activated protein kinase; MKI67, marker of proliferation Ki-67; MMP-2, matrix metallopeptidase 2; NCOR2, nuclear receptor corepressor 2; NDUF3,NADH dehydrogenase [ubiquinone] 1 alpha subcomplex assembly factor 3; NDUFS3, mitochondrial NADH dehydrogenase [ubiquinone] iron-sulfur protein 3; NDUS7, NADH dehydrogenase iron-sulfur protein 7; OXPHOS, oxidative phosphorylation; PI3K, phosphatidylinositol 3-kinase; PTEN, phosphatase and tensin homolog; QKI-6, Quaking gene isoform 6; RON, macrophage stimulating 1 receptor, MST1R; ROS, reactive oxygen species; RRM2, ribonucleotide reductase regulatory subunit M2; SOX2, SRY-box transcription factor 2; STAT3, signal transducer and activator of transcription 3; TMZ, temozolomide; WWOX, WW domain containing oxidoreductase.

Each modification has its writers, the enzymes that deposit RNA chemical marks; erasers, the enzymes that remove these marks; and readers, the proteins that selectively recognize and bind to these marks (Figure 1). Collectively, the writers, erasers and readers also refer to as RNA-modifying proteins (RMPs). These modifications are not only needed for normal function but also play essential roles in pathological processes, such as tumorigenesis.

## 3. RNA Modifications in GBM

With the developing of sequencing technology in RNA modifications, novel markers regarding RNA modifications in cancers are also emerging [34]. GBM is the most malignant tumors in the brain that has no effect therapy. Therefore, it is urgent to develop new strategies to treat this disease. Since m^6^A is the best-known RNA mark, it is highly connected with GBM progression and aggressiveness and some regulators may be potential drug targets. Recently, other kinds of RNA marks, including m^6^A_m_, m^1^A, m^5^C, hm^5^C, I and ψ, as well as their modulators are also emerging to be correlated with GBM progression (Table 1).

### 3.1. RNA m^6^A Modification in GBM

RNA m^6^A modification is the most prevalent and abundant modifications that occur in the mRNAs, rRNAs and small nuclear RNAs (snRNAs) [35]. m^6^A modification of mRNA usually occurs in nuclear speckles where the methyltransferases and demethylases are concentrated [29] and are enriched in single nucleotide polymorphisms (SNPs) [36]. Generally, the enzymatic core METTLE3 and methyltransferase like 14 (METTL14), as well as Wilms’ tumor 1-associated protein (WTAP), Virilizer like m6A methyltransferase associated protein (VIRMA/KIAA1429), RNA-binding motif protein 15 (RBM15), RNA-binding motif protein 15B (RBM15B/OTT3), zinc finger CCCH domain-containing protein 13 (ZC3H13, as known as Flacc and KIAA0853) and Hakai (also known as Cbl proto-oncogene like 1, CBLL1) constitute of a methyltransferase complex, also known as m^6^A-METTL associated complex (MACOM), that can mediates the m^6^A methylation. Meanwhile, two demethylases, Fat mass and obesity-associated protein (FTO) and ALKBH5, catalyze the m^6^A demethylation [37,38,39,40,41,42].

Several m^6^A-binding proteins such as YTH N^6^-methyladenosine RNA binding protein 1/2/3 (YTHDF1/2/3) and YTH domain-containing 1/2/3 (YTHDC1/2/3), which have the YTH domain, are readers that mediate the degradation of m^6^A-labelled RNAs [43,44]. Other factors, such as insulin-like growth factor 2 mRNA-binding protein 1/2/3 (IGF2BP1/2/3, also known as IMP1/2/3), eukaryotic translation initiation factor 3a/b/h (eIF3a/b/h), heterogeneous nuclear ribonucleoprotein A2/B1 (hnRNPA2B1) and heterogeneous nuclear ribonucleoprotein C1/C2 (hnRNPC) were recently shown to be able to read the m^6^A marks, too [22]. These m^6^A reader proteins can recognize these marks and also can play key roles in the RNA stabilization of the writers [45]. m^6^A marks recognized by different readers with different sub-locations may exert different functions: the readers in the nucleus, such as hnRNPC, hnRNPA2B1 and YTHDC1, are responsible for RNA structure switching, alternative splicing, microRNA maturation, RNA stability, RNA export and X chromosome inactivation; while readers in the cytoplasm, such as YTHDF1/2/3 and YTHDC2, are mainly responsible for mRNA translation and decay [46].

As RNA m^6^A affects RNA stability, processing, splicing, translation, and the epigenetic function of some non-coding RNAs (ncRNAs) [47,48], it plays essential role in cell fate determination, embryonic stem cell maintenance and specification, T-cell homeostasis, neuronal functions, sex determination as well as pathogenesis [49,50,51,52,53,54]. In tumors, m^6^A also emerges as an important modulator. For instance, METTL3 drives tumorigenicity and metastasis through suppressing suppressor of cytokine signaling 2 (SOCS2) expression in hepatocellular carcinoma [55].

Actually, m^6^A methylomes of brain tissues are highly specific [35], but the status of m^6^A may be different in GBM. A bioinformatic analysis in The Chinese Glioma Genome Atlas (CGGA) microarray and RNA sequencing databases [58] show that m^6^A writers, including METTL3, METTL14, WTAP, RBM15 and RBM15B, as well as m^6^A readers including YTHDC2, YTHDF1, YTHDF2, YTHDF3, hnRNPA2B1 and hnRNPC are elevated, while m^6^A writer ZC3H13 and m^6^A eraser FTO are decreased in gliomas. In GBMs, m^6^A writers WTAP and RBM15, m^6^A eraser ALKBH5, and m^6^A reader YTHDF2 are significantly elevated, while m^6^A writers METTL3, VIRMA and ZC3H13, m^6^A eraser FTO, m^6^A readers YTHDC2 and hnRNPC are significantly decreased, compared with low-grade gliomas (LGGs). In addition, four lncRNAs including MIR9-3HG, LINC00900, MIR155HG, and LINC00515 seem to be related with m^6^A. Another bioinformatic analysis [92] revealed that the expressions of WTAP, RBM15, YTHDF and ALBKH5 are positively, while FTO expression is negatively associated with WHO grades of glioma. Additionally, expression levels of ALKBH5, YTHDF2, RBM15, METTL3, METTL14, FTO and YTHDC1 in LGGs with or without mutant isocitrate dehydrogenase (IDH) are significantly different. Moreover, FTO, YTHDC1 and METTL3 are also differentially expressed between GBM with and without IDH mutation. Importantly, the expressions of gliomas malignant clinicopathological features-related m^6^A RNA methylation regulators are tightly correlated with pro-oncogenic genes in gliomas. These evidences imply that m^6^A modifications are tightly correlated with GBM progression. According to present studies in GBM, m^6^A modification seems to play pivotal roles since both m^6^A writers and erasers contribute to the tumorigenesis of GBM (Figure 1), especially glioma stem-like cells (GSCs).

#### 3.1.1. RNA m^6^A Writers in GBM

The expression of METTL3, the most important m^6^A writer, is significantly elevated in GSCs, while it is attenuated during GSCs differentiation [56]. Elevated expression of METTL3 are associated with the clinical aggressiveness of malignant gliomas [57]. An integrated analysis of m^6^A-RNA immunoprecipitation (RIP) and total RNA-Sequencing (RNA-seq) of METTL3-silenced GSCs revealed that METTL3 is the most important writer responsible for m^6^A modification in GSCs [45]. Moreover, METTL3 silencing also reverses RasV12-mediated malignant transformation of mouse immortalized astrocytes and suppresses GBM tumor growth in vitro and in vivo [56,57]. Furthermore, METTL3 silencing in GSCs enhances their sensitivity to γ-irradiation and reduces DNA repair [56]. Mechanically, METTL3 seems to be responsible for the m^6^A modification in some GSCs-specifically-expressed genes [45]. Particularly, METTL3 mediates m^6^A modification of mRNA of SRY-box transcription factor 2 (SOX2), a pluripotent gene, at three METTL3/m^6^A sites present in its 3′ untranslated region (UTR), leading to the recruitment of human antigen R (HuR) to SOX2 mRNA to enhance its stability, thereby resulting in GSC maintenance and dedifferentiation [56].

In addition to directly affecting the pluripotent genes, an m^6^A-specific methylated RNA immunoprecipitation with NGS (MeRIP-Seq or m^6^A-Seq) analysis also shows that m^6^A modification peaks seem to be enriched at transcripts responsible for metabolic pathways in GSCs, compared with neural progenitor cells [57]. METTL3 also causes alterations such as decrease of adenosine-to-inosine (A-to-I) and cytidine to uridine (C-to-U) RNA editing events in GSCs by downregulating adenosine deaminase RNA specific 1 (ADAR1) and apolipoprotein B mRNA editing enzyme catalytic subunit 3A (APOBEC3A), which are RNA editing enzymes [45]. Furthermore, gene ontology analysis reveals that the direct targets of METTL3 seems to be enriched in some major oncogenic pathways including NOTCH signaling, vascular endothelial growth factor (VEGF) signaling, angiogenesis, glycolysis and Hedgehog signaling pathways, whereas the indirect targets are enriched in the RAS, mitogen-activated protein kinase (MAPK), G protein-coupled receptors (GPCRs), cadherin signaling pathways and cell cycle [45]. Additionally, in GSCs, METTL3-dependent m^6^A modification also affects the levels of serine- and arginine-rich splicing factors (SRSFs) through upregulating BCL-X (Bcl-2-like protein 1, BCL2L1) or nuclear receptor corepressor 2 (NCOR2) isoforms, which are important to prevent YTHDC1-dependent nonsense-mediated mRNA decay (NMD) [57]. lncRNAs with METTL3-dependent m^6^A marks is also highly expressed in GSCs, compared with that of protein-coding genes [45]. m^6^A marks within 3′UTRs seem to hinder the miRNA binding in GSCs [45].

Nevertheless, an opposite study shows that METTL3 and METTL14 may be a tumor suppressor in GBM [59]. Their results show that deficiency of METTL3 or METTL14 significantly enhances the growth, self-renewal, and tumorigenesis of GBM stem cells. Subsequently, m^6^A sequencing and molecular experiments implies that METTL3 or METTL14 knockdown induced alterations in mRNA m^6^A enrichment and the mRNA expression of critical factors, such as a disintegrin and metallopeptidase domain 19 (ADAM19), that participate in modulation of GBM stem cells. In contrast, overexpression of METTL3 renders GBM stem cells growth and self-renewal. Another study in established GBM cell line U251 cells also shows the similar results: METTL3 overexpression reduces migration and proliferation ability and induces cellular apoptosis possible by downregulating heat shock protein 70 (HSP70) expression [60]. The controversial conclusion may be due to the samples they used. These results mean that function of m^6^A modifications regulated by METTL3 in GBM should indeed be further confirmed.

WTAP is also an important part of the m^6^A methyltransferase complex. It is also highly expressed in glioma, closely correlated with pathological grade and negatively correlated with postoperative survival in glioma patients [61,62]. WTAP stimulates tumorigecity, migration and invasion of GBM cells possibly through enhancing the activity of epidermal growth factor receptor (EGFR), an important oncogenic factors in GBM [61]. In GSCs, WTAP also plays an important role as a downstream target of miR-29a/Quaking gene isoform 6 (QKI-6) axis-mediated inhibition of cell proliferation, migration and invasion, as well as promotion of apoptosis in GSCs [63].

ZC3H13, as a zinc-finger protein, is also an essential regulator in the MACOM complex, which can anchor WTAP, Virilizer, and Hakai in the nucleus [93]. A recent study shows that ZC3H13 mutation combined with retinoblastoma 1 (RB1) mutation can recapture human GBM in a mouse model. Moreover, ZC3H13 mutation also alters the gene expression profiles of RB1 mutants, rendering GBM tumors more resistant to TMZ [94]. These evidences connote that ZC3H13 may be an anti-cancer factor.

Collectively, m^6^A writers are important for GBM progression. Most of them are upregulated in GBM, and show oncogenic roles by regulating specific signaling pathways, especially factors that contribute to stemness maintenance. However, some opposite results show that the expression alterations of some writers in GBM are downregulated and they may pose anti-cancer properties, indicating that much more work should be done to confirm their expressions and functions in GBM.

#### 3.1.2. RNA m^6^A Erasers in GBM

Like the writers, m^6^A erasers also play essential roles in GBM. Inhibition of FTO, an m^6^A eraser, also impedes the self-renewal ability and tumorigenecity of GBM stem cells both in vitro and in mice models [59]. Other studies also show that FTO functions as an oncogenic role via maintaining the stability of transcripts of avian myelocytomatosis viral oncogene homolog (c-Myc) and CCAAT enhancer binding protein alpha (CEBPA) in glioma, especially IDH1/2 mutant glioma [64]. Besides, inhibition of FTO by its specific inhibitor, MA2, significantly renders the tumorigenecity in GSC-grafted mice [59]. These evidences suggest that the homostasis of m^6^A modification in cells is important and FTO may be a promising drug target of GBM.

Another m^6^A demethylase, ALKBH5, is also highly expressed in patient-derived GSCs. Silencing ALKBH5 inhibits cell proliferation of GSCs. An integrated transcriptome and m^6^A-seq analysis identified several ALKBH5 target genes, including forkhead box M1 (FOXM1), a transcription factor that is critical for GSCs. ALKBH5 demethylates FOXM1 nascent transcripts, thereby enhancing its expression. A lncRNA forkhead box M1-antisense RNA (FOXM1-AS), also enhances the interaction of ALKBH5 with FOXM1 nascent transcripts [65]. These results show that m^6^A erasers may also play pro-oncogenic roles in GBM and may be potential drug targets for GBM treatment. If both m^6^A writers and erasers are confirmed to be upregulated in GBM, it is probable that the modulatory capacity of m^6^A in GBM is enhanced, indicating that GBM may be more flexible to response to complex extracellular or intracellular stresses.

#### 3.1.3. RNA m^6^A Readers in GBM

The fate of m^6^A-modified RNA is determined by m^6^A readers, so they may be more important factors for cancer treatment, because when functions of m^6^A writers and erasers are settled, the functions of readers depend on what kinds of readers to carry out the effects of m^6^A marks. Importantly, the aberrant expression of m^6^A readers may contribute to GBM tumorigenesis independently of m^6^A alterations to some extent. This means that even in the condition that m^6^A is not changed, the effect of m^6^A may be altered by the abnormal expression of m^6^A readers. Based on current study, several families that exert as m^6^A readers are tightly related to the tumorigenesis of GBM.

##### YTHDF and YTHDC Families

YTHDF and YTHDC families are the most important m^6^A readers that contain an YTH domain that can bind to RNAs. They exhibit different functions: YTHDC1 mediates mRNA splicing; YTHDF2, YTHDF3, and YTHDC2 mediate mRNA decay; YTHDF1, YTHDF3, and YTHDC2 mediate translation; and YTHDC2 mediates RNA structures [22]. YTHDC1 reads the m^6^A marks dependent on its tryptophan 377 (W377) or W428 sites [95]. Recently, these readers were shown to also participate in tumorigenesis of GBM. For instance, YTHDC1 deficiency reduces sphere number substantially in METTL3 overexpressed U87 cells but not in control cells. Overexpressing W377A/W428A mutant YTHDC1 cannot promotes the sphere formation capacity of U87 cells, suggesting that YTHDC1 promotes GBM phenotype dependently on its m^6^A binding activity [57].

##### IGF2BP Family

Another m^6^A reader protein family, the IGF2BP family, including IGF2BP1/2/3, inhibits the degradation of m^6^A-modified transcripts and facilitates their translation [96]. For a long time, these factors are recognized as important regulators in GBM progression, even though there are no direct connections between their functions in m^6^A readers and oncogenic roles in GBM. For example, IGF2BP1 promotes cell proliferation and invasion in GBM cells through stabilizing the mRNA transcripts of its target genes, including c-Myc, marker of proliferation Ki-67 (MKI67), phosphatase and tensin homolog (PTEN) and CD44 [66,67]. However, whether this phenomenon depens on its activity in m^6^A reading remains unknown.

IGF2BP2 is also upregulated in GBM tissues. It supports GBM cell proliferation, migration, invasion and epithelial-to-mesenchymal transition (EMT) via regulating the activity of insulin-like growth factor 2 (IGF2), which further activates phosphatidylinositol 3-kinase (PI3K)/Akt signaling. Additionally, inhibition of IGF2BP2 sensitizes GBM to TMZ treatment [68]. Another study shows that IGF2BP2 binds to let-7 miRNA recognition elements (MREs) and prevents LIN28-independent let-7 target gene silencing, including high mobility group AT-hook 1 (HMGA1), HMGA2, cyclin D2 (CCND2) and ribonucleotide reductase regulatory subunit M2 (RRM2), thereby ensuring maintenance of stemness in GSCs [69]. In this process, IGF2BP2 is responsible for miRNA maturation. IGF2BP2 also interacts with lncRNAs such as hypoxia-inducible factor 1 α-antisense RNA 2 (HIF1A-AS2), which functions as a subtype-specific hypoxia-inducible lncRNA, thereby maintaining the expression of their target gene, HMGA1, resulting in promoted GSC growth, self-renewal, and hypoxia-dependent molecular reprogramming [71]. In addition, IGF2BP2 also interacts with mRNAs and even proteins. For example, it can promote oxidative phosphorylation (OXPHOS) in primary GBM sphere cultures (gliomaspheres, GSCs) via binding several mRNAs such as mitochondrial cytochrome c oxidase subunit 7B (COX7B), NADH dehydrogenase iron-sulfur protein 7 (NDUS7), NADH dehydrogenase [ubiquinone] 1α subcomplex assembly factor 3 (NDUF3) and mitochondrial NADH dehydrogenase [ubiquinone] iron-sulfur protein 3 (NDUFS3), that encode mitochondrial respiratory chain complex subunits. Additionally, it also interacts with complex I (NADH: ubiquinone oxidoreductase) proteins such as NDUFS3, thereby promoting clonogenicity in vitro and tumorigenicity in vivo [70].

IGF2BP3 mRNA and protein levels are also upregulated in GBMs but not in lower grade astrocytomas [97]. Particularly, IGF2BP3 is one of the genes overexpressed in pilomyxoid astrocytomas (PMAs) compared with a less aggressive pilocytic astrocytomas (PAs), according to a gene expression microarray analysis of 9 PMAs and 13 PAs from infra- and supratentorial sites [98]. A microarray analysis in glioma cells shows that IGF2BP3 mediates regulation of direct targets at transcriptome associated with cell cycle process, whereas direct targets at the translatome participating in apoptosis related pathways [99]. IGF2BP3 also promotes cell proliferation, migration and invasion by inducing EMT via downregulating the expression of E-cadherin and upregulating the expressions of N-cadherin, vimentin, snail, slug and MMP-9 in GBM [100]. In addition, IGF2BP3 promotes cell proliferation, anchorage-independent growth, invasion, and chemoresistance via activating PI3K/MAPK pathways by binding to the 5′-UTR of IGF-2 mRNA to activate its translation [97]. IGF2BP3 also stimulates glioma cell migration by enhancing the translation of p65 (RELA), a subunit of nuclear factor kappa-B (NF-κB) heterodimer, which can also in turn transcriptionally activates IGF2BP3 to form a feedback loop [101].

##### eIF3 Family

eIF3a/b/h, also function as m^6^A readers, and can directly, physically and functionally interact with METTL3, thereby enhancing translation, and the formation of densely packed polyribosomes through recognizing m^6^A modification at the 5′ UTR of mRNA [102,103,104]. A study of cancer predisposition syndromes (CPS) shows that eIF3h is duplicated in a pediatric patient with infantile desmoplastic astrocytoma and low-grade glioma [73]. eIF3b silencing significantly inhibits cell proliferation of U87 cells via inducing G0/G1-phase arrest and apoptosis [72]. These evidences indicate that eIF3 family members may play specific roles in GBM.

##### hnRNPC and hnRNPA2B1

During nuclear RNA processing period, hnRNPC and hnRNPA2B1 are other types of m^6^A readers that can bind to unfolded RNA through an m^6^A structural switch mechanism, since the m^6^A marked RNA cannot efficiently form secondary structures because the base pairing of m^6^A-U is weaker of than that of A-U [105,106]. A study on the SOX2 protein-protein interactome reveals that hnRNPC and hnRNPA2B1 can interact with SOX2 in GBM [107], suggesting that they may play key roles in maintaining the stemness of GSCs.

Actually, hnRNPC is one of the important physiological and cancer-related regulators (including GBM) of 3′ UTR processing and miRNA maturation [108]. hnRNPC has a higher abundance in higher grade GBM. Mechanically, hnRNPC directly binds to primary mir-21 (pri-mir-21) and promotes the processing of miR-21 that targets programmed cell death 4 (PDCD4), an important regulator of apoptosis and cellular survival. PDCD4 subsequently supports Akt and p70 S6 kinase (p70S6K) activation, and then promotes migratory and invasive activities, increases cell proliferation and protects GBM cells from etoposide-induced apoptosis [76].

hnRNPA2B1 is also an important modulator of pre-mRNA processing, mRNA metabolism and transportation in cells. hnRNPA2B1 protein is highly expressed in glioma tissues, correlated with glioma grades and related to poor prognosis [75]. Mechanically, hnRNPA2B1 promotes GBM cell viability, adhesion, migration, invasion, chemoresistance to TMZ and tumorigenecity as well as protects cells from apoptosis and reactive oxygen species (ROS) generation possibly via downregulating tumor suppressors such as cellular FLICE (FADD-like IL-1β-converting enzyme)-inhibitory protein (c-FLIP), bridging integrator 1 (BIN1) and WW domain containing oxidoreductase (WWOX), and upregulating phospho-signal transducer and activator of transcription 3 (p-STAT3), matrix metallopeptidase 2 (MMP-2), and the proto-oncogene RON (also known as macrophage stimulating 1 receptor, MST1R) [74,75]. These results indicate that hnRNPC and hnRNPA2B1 are possible m^6^A readers that contribute to GBM pathogenesis.

Collectively, from the current study, YTHDC1 is the only confirmed m6A reader that exerts its function in GBM dependently on its biological function in m^6^A RNA modification. Whether other readers’ functions are m^6^A-dependent remains unknown. However, most of their functions are dependent on their RNA binding capacity, implying that m^6^A in RNAs may be involved in their binding and their functions in GBM. This hypothesis needs to be further confirmed. As an important mark, m^6^A may have more readers than we can imagine. These readers are like messengers of m^6^A marks but beyond that, because they not only deliver message of m^6^A modified mRNA, but also determine their fate.

### 3.2. RNA m^6^A_m_ Modification in GBM

N^6^,2′-O-dimethyladenosine (m^6^A_m_) is a terminal modification at mRNA cap (usually occurs in the first and sometimes the second nucleotide after the N^7^-methylguanosine (m^7^G) cap) in higher eukaryotic mRNAs and ncRNAs, expecially snRNAs, as well as viral RNAs [109]. m^6^A_m_ plays essential roles in RNA splicing [110], snRNA biogenesis [111], mRNA stability [112] and cap-dependent translation [113]. In human beings, phosphorylated CTD interacting factor 1 (PCIF1, also known as cap-specific adenosine methyltransferase, CAPAM) and methyltransferase-like 4 (METTL4) are the m^6^A_m_ writers, the m^6^A eraser FTO also functions as an m^6^A_m_ eraser, and mRNA-decamping enzyme 2 (DCP2) may be one of the m^6^A_m_ readers [109,110,111,114,115].

**Figure 2:**
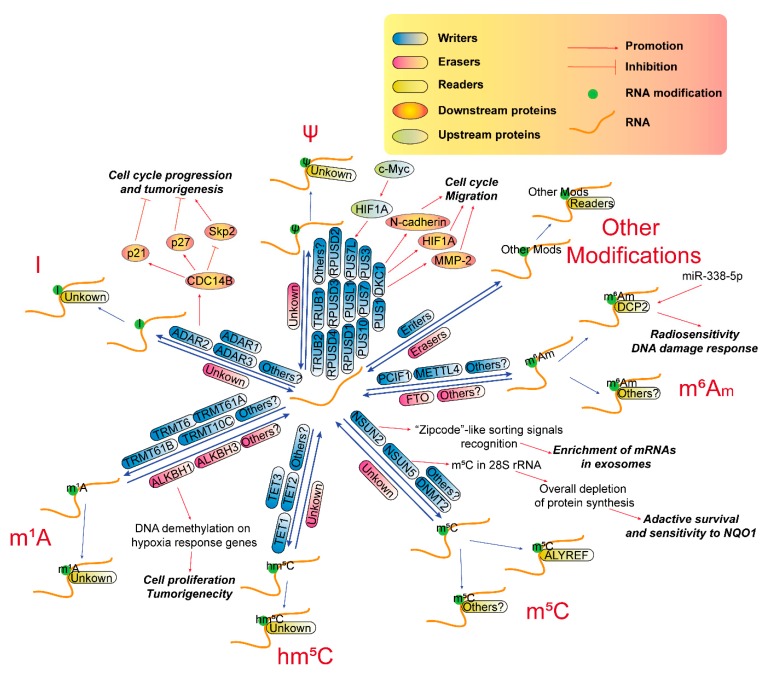
Emerging functions of RNA modifications (except m^6^A) and their writers, erasers and readers in GBM. Abbreviations: Modifications: A-to-I, adenosine-to-inosine; m1A, N1-methyladenosine; m5C, 5-methylcytosine; m6Am, N6, 2′O-dimethyladenosine; hm5C, 5-hydroxymethylcytosine; I, inosine; ψ, pseudouridine. RNA-modifying proteins (RMPs): ADAR1/2/3, adenosine deaminase that acts on RNA 1/2/3; ALYREF, ALY/REF export factor; DCP2, mRNA- decapping enzyme 2; DKC1, dyskerin pseudouridine synthase 1; DNMT2, DNA (cytosine-5-)-methyltransferase 2; METTLE4, methyltransferase like 4; NSUN2/5, NOP2/Sun RNA methyltransferase 2/5; PCIF1, phosphorylated CTD interacting factor 1; PUS1/3/7/10, pseudouridine synthase 1/3/7/10; PUSL1, pseudouridine synthase like 1; PUS7L, pseudouridine synthase 7 like; RPUSD1/2/3/4, RNA pseudouridylate synthase domain-containing 1/2/3/4; TET1/2/3, ten-eleven translocation (TET) family; TRMT6/61A, tRNA methyltransferase 6/61A; TRMT61B, tRNA methyltransferase 61 homolog B; TRMT10C, tRNA methyltransferase 10C, mitochondrial RNase P subunit; TRUB1/2, TruB pseudouridine synthase family member 1/2. Others: c-Myc, avian myelocytomatosis viral oncogene homolog; CDC14B, cell division cycle 14B; NQO1, Quaking gene isoform 6; HIF1A, hypoxia-inducible factor 1 alpha; Mods, modifications; p70S6K, p70 S6 kinase;Skp2, S-phase kinase associated protein 2; PDCD4, programmed cell death 4; Skp2, S-phase kinase associated protein 2.

Recent evidence shows that FTO-mediated m^6^A_m_ demethylation is an important regulatory mechanism in adjusting the stem-like properties of colorectal cancer cells [116], and many clues also imply that m^6^A_m_ also emerges as an essential regulator in GBM (Figure 2, m^6^A_m_ part). For instance, an integrated proteogenomic analysis in 17 GBM patient samples reveals that METTL4 is one of the top-ranked missense mutation genes [77]. DCP2 is upregulated in miR-338-5p overexpressed GBM cells and may participate in radiosensitivity and DNA damage response induced by miR-338-5p overexpression in GBM cells [78]. DCP2 is also one of top 20 hub genes of the optimal competing endogenous RNA (ceRNA) network in GBM and other cancers [117]. These results imply that m^6^A_m_ probably participates in the development of GBM.

### 3.3. RNA m^5^C Modification in GBM

5-Methylcytosine (m^5^C) in DNA has been well described, however, it is not well understood in RNAs. Actually, m^5^C is not only confined within tRNAs and rRNAs, but also present in mRNA and ncRNAs [118]. RNA m^5^C usually responsible for stabilization of tRNA molecules via affecting Mg^2+^ binding, regulating mRNA translation, preventing recognition of endogenous RNAs by the innate immune system, and modulating RNA-dependent inheritance of certain phenotypes [119]. In human beings, NOP2/Sun RNA methyltransferase 2 (NSUN2, Misu), NSUN5 and DNA (cytosine-5-)-methyltransferase 2 (DNMT2) are confirmed as m^5^C writers, while ALY/REF export factor (ALYREF) is an m^5^C reader [119].

As a target of proto-oncogene c-Myc [120], NSUN2 has been shown to play pivotal roles in many neoplasms, such as breast cancer [121], colorectal cancer [122], ovarian cancer [123], head and neck squamous carcinoma [124] as well as esophageal squamous cell carcinoma [125]. In GBM, there is no direct evidences about the biological function of NSUN2. However, “zipcode”-like sorting signals in RNA that mediate the enrichment of mRNAs in exosomes of GBM cells [126] may probably be recognized by cytosolic Y-box binding protein 1 (YB-1) and NSUN2 [127]. NSUN5, another RNA methyltransferase that is responsible for m^5^C in the C3782 position of human 28S rRNA, undergoes epigenetic loss in gliomas, which drives an overall depletion of protein synthesis, resulting in an adaptive translational program for survival under cellular stress and renders gliomas sensitive to bio-activatable substrates of the stress-related enzyme NAD(P)H quinone dehydrogenase 1 (NQO1) [81,82]. The m^5^C reader, ALYREF, may be an overexpressed antigen in GBM, according to a study with a nanobody-based anti-proteome approach [83] and is upregulated in recurrent GBM, according to a transcriptome analysis [84], implying it may be a promising drug target for GBM. These evidences show m^5^C and its RMPs may also play important roles during the tumorigenesis of GBM (Figure 2, m^5^C part), indicating they are promising pharmaceutical targets for GBM treatment.

### 3.4. RNA hm^5^C Modification in GBM

In mammals, both DNA and RNA m^5^C are subject to oxidative processing by the ten-eleven translocation (TET) family (TET1/2/3), forming 5-hydroxymethylcytidine (hm^5^C) and 5-formyl-cytidine (f^5^C), which are considered as mediating substances during the demethylation pathway [128]. However, a recent study shows that m^5^C oxidation is a conserved process that could have critical regulatory functions inside cells [129]. For example, hm^5^C is distributed at lncRNA loci, involved in long-range chromatin interactions and positively correlated with lncRNA transcription [130]. TET1 is elevated in GBM [85], while TET2 expression is downregulated in glioma [86], and TET3 is epigenetically repressed in glioma [87]. 5-hydroxymethylcytidine in DNA has been recognized as important markers in GBM [131,132,133], however, the direct evidence of hm^5^C modification in RNA has never been explored in GBM (Figure 2, hm^5^C part).

### 3.5. RNA m^1^A Modification in GBM

N^1^-methyladenosine (m^1^A) is a reversible modification in tRNA, mRNA and lncRNAs, even in mitochondrial transcripts, and is critical for tRNA stability, and translation of mitochondrial DNA-encoded proteins [134]. In nucleus, m^1^A modifications in pre-tRNA and pre-mRNA are catalyzed by tRNA methyltransferase 6/61A (TRMT6-TRMT61A) catalytic complex and are erased by ALKBH1 and ALKBH3, respectively [135,136]. Whereas in mitochondrion, m^1^A methylation in mt-tRNA and mt-mRNA are installed by tRNA methyltransferase 61 homolog B (TRMT61B) and tRNA methyltransferase 10C, mitochondrial RNase P subunit (TRMT10C) [137]. Recently, some RMPs of m^1^A, both writers and erasers, seem to involve in the progression of GBM (Figure 2, m^1^A part). TRMT6 and TRMT61A expression is significantly upregulated in highly aggressive GBM compared with grade II/III gliomas [79]. TRMT61A is a target of HIF1A and is dowregulated after c-Myc inhibition in GBM under hypoxia [138]. The eraser ALKBH1 also seems to be an important regulator in GBM, since targeting ALKBH1 in patient-derived GBM models induces cell proliferation inhibition and extends the survival of tumor-bearing mice. However, this effect may be caused by ALKBH1-induced DNA demethylation on hypoxia response genes, while not ALKBH1-controlled tRNA stability [80].

### 3.6. RNA A-To-I Modification in GBM

A-to-I RNA editing is abundant in the human transcriptome and plays essential roles in RNA processing, such as post-transcriptionally altering codons, introducing or removing splice sites, and affecting the base pairing of the RNA molecule with itself or with other RNAs [139]. All 3 editing mediating enzymes, ADAR1 (ADAR), ADAR2 (ADARB1), and ADAR3 (ADARB2), is downregulated in brain tumors and ADARB2 is also negatively correlated with GBM grades. ADAR1 and ADAR2 overexpression in GBM cell line inhibit cell proliferation, suggesting that reduced A-to-I editing contributes to the pathogenesis of this disease [88]. In addition, abnormal expression of an ADAR2 alternative splicing variant also downregulates A-to-I RNA editing in glioma [89], because ADAR2 can prevent GBM tumor growth via modulating an important cell cycle pathway involving S-phase kinase associated protein 2 (Skp2), p21 and p27 proteins by introducing an A-to-I editing in the pre-mRNA of a phosphatase cell division cycle 14B (CDC14B) [90]. Additionally, A-to-I editing in miR-376a-3p is attenuated in GBM, thus promoting invasiveness of this disease [140]. These results show that A-to-I editing is essential for GBM progression (Figure 2, I part), and RMPs of A-to-I modification may be potential targets for GBM treatment.

### 3.7. RNA ψ Modification in GBM

Pseudouridine (ψ), the most abundant RNA modification, is occurs in almost all kinds of RNAs, including tRNA, mRNA, snRNA, nucleolar RNA (snoRNA), and rRNA [141,142]. Ψ is important for rRNA folding, translational fidelity and rate, stress response, tRNA codon–anticodon base-pairing, snRNP biogenesis and pre-mRNA splicing [143]. There are 13 pseudouridine synthases (PUSs, writers) including PUS1, PUS3, PUS7, PUS10, pseudouridine synthase like 1 (PUSL1), pseudouridine synthase 7 like (PUS7L), TruB pseudouridine synthase family member 1 (TRUB1), TRUB2, RNA pseudouridylate synthase domain-containing 1 (RPUSD1), RPUSD2, RPUSD3, RPUSD4 and dyskerin pseudouridine synthase 1 (DKC1) [144,145]. Recently, emerging evidence also shows that ψ may play pivotal roles during gliomagenesis. One of the ψ writers, DKC1, is significantly upregulated in glioma and correlates with the WHO stages of tumors. DKC1 promotes glioma cell growth by stimulating cell cycle progression and activates migration via upregulating N-cadherin, hypoxia inducible factor 1 subunit α (HIF1A), and MMP-2 expression [91]. PUS7L, as a target of HIF1A, is also downregulated after c-Myc inhibition in GBM under hypoxia [138]. These emerging evidences provide us the importance of pseudouridine modification in RNAs during the pathogenesis of GBM (Figure 2, ψ part).

### 3.8. Other RNA Modifications in GBM

Although most of the other RNA modifications are not well described, there are also some studies regarding the effect of these RNA modifications on GBM progression. For instance, ADAR2 and ADAR3 binds to the pre-mRNA of glutamate receptor subunit B, glutamate receptor ionotropic AMPA (GRIA2), contributing to a RNA editing at the Q/R site by modifying a codon replacing the glutamine (Q) with arginine (R). ADAR2/3 deficiency in GBM leads to increased unedited GRIA2 subunits, thereby leads to a calcium-permeable glutamate receptor, which can promote cell migration and tumor invasion [146]. Cleavage factor Im 25 (CFIm25) restricts mRNA proximal poly(A) site usage via alternative polyadenylation (APA). CFIm25 knockdown leads to at least 1,450 genes with shortened 3′UTRs and marked increases in the expression of several oncogenes in GBM, including cyclin D1 (CCND1), glutaminase (GLS) and methyl-CpG-binding protein 2 (MECP2), thereby enhancing their tumorigenesis [147]. Mechanically, shortened 3′UTR truncation of oncogenic transcripts relieves intrinsic microRNA- and AU-rich-element-mediated repression. These new findings unravel a wide horizon for us to explore the implements of RNA modifications in cancer treatment. With the developing technology, much more modifications in RNA and their RMPs, as well as their connections with human diseases, especially cancers, will be disclosed.

## 4. Potential Clinical Implications of RNA Modifications in GBM

The descriptions above indicate that RNA modifications are important for tumorigenesis and aggressiveness in GBM. Chemical modifications in both DNA and proteins have been well studied in cancer biology, and many chemical modifications such as DNA methylation, histone modification and EGFR post-translational modifications, as well as some of their modification enzymes have been shown to have clinical significance in both the diagnosis and therapy [148,149,150,151,152,153,154,155]. Importantly, these chemical modifications are also important for GBM diagnosis and therapy [8,156,157,158]. Actually, as kinds of chemical modifications, RNA modifications and their RMPs also give promise to both the diagnosis and therapeutics of GBM in the future.

### 4.1. Potential Diagnostic Implications of RNA Modifications in GBM

Epigenetic alterations are considered as promising markers for GBM diagnosis [159,160,161,162], although this study is just the first step for their possible applications in GBM diagnosis. Besides, some epigenetic status really account for the outcome of GBM. For example, the epigenetic silencing of the O^6^-methylguanine–DNA methyltransferase (MGMT) significantly affects the TMZ treatment of GBM [163]. Therefore, MGMT promoter methylation status is an important biomarker to predict survival and TMZ response in patients with GBM [164].

As a kind of chemical modifications in RNA, the alterations of their regulations may also predict the prognosis or be used for diagnosis of GBM. Actually, m^6^A in in peripheral blood RNA has shown to be a predictive biomarker for gastric cancer [165]. Methylation levels in miRNAs are also potential diagnostic biomarkers for early-stage gastrointestinal cancers [166]. The m^6^A erasers ALKBH5 and FTO are also shown to be prognostic biomarkers in patients with clear cell renal carcinoma [167]. From Table 1, some RNA modification enzymes are also tightly related to the grades and may predict the prognosis of GBM. However, the specificity of their expression should be further confirmed. Besides, some RNA modifications in peripheral blood RNA may also be promising methods for GBM diagnosis. In the future, it can be suspected that RNA modification marks as well as their RMPs’ expression may be important biomarkers for the diagnosis of GBM.

### 4.2. Potential Therapeutical Implications of RNA Modifications in GBM

Covalent modifications in both DNA, histones and other proteins have shown promising probability for cancer treatment. Actually, some epigenetic drugs such as azacitidine, 5-aza-2′-deoxycytidine, suberoylanilide hydroxamic acid (SAHA), romidepsin, belinostat, panobinostat, has been approved by the United States Food and Drug Administration (FDA) and chidamide has been approved by China FDA to treat some T-cell lymphoma or multiple myeloma, and many other epigenetic drugs on cancers are ongoing clinical trials [10]. Besides, drugs that target modulators of protein post-translational modifications, such as E3 ubiquitin ligases including mouse double minute 2 homolog (MDM2), sphase kinaseassociated protein 2 (SKP2), speckle type BTB/POZ protein (SPOP), cellular inhibitor of apoptosis 2 (cIAP) and anaphase-promoting complex/cyclosome (APC/C), are also been evaluated in clinical and preclinical settings [168]. Importantly, some epigenetic drugs such as vorinostat, panobinostat, romidepsin and valproic acid alone or in combination with other chemotherapeutic methods such as TMZ treatment or radiotherapy are studying in phase I/II clinical trials [169].

As kinds of covalent modifications in RNA, their potentials in cancers like malignant GBM are being explored. Since more than 170 kinds of modifications, it is a wider world for us to explore. Specific antagonist against writers, erasers and readers of RNA modifications needs to be screened, designed and identified, as well as their biological functions and clinical applications in cancer treatment also need to be extensively studied. In fact, several pharmaceutical companies are planning to develop small-molecule inhibitors of the METTL3–METTL14 complex and ADAR [170]. Several compounds with piperidine and piperazine rings have exceptionally high docking efficiencies to METTL3-METTL14-WTAP complex, thereby activating RNA methylation [171]. Recently, a small molecule inhibitor of METTL3 is also shown to curb the development of acute myeloid leukemia (AML) in vivo [172,173]. We believe that effective and efficient therapies regarding RNA modifications will soon be developed to treat malignant neoplasms such as GBM.

## 5. Conclusions and Perspectives

From the collective evidence, we can conclude that RNA modifications play increasingly important roles in the tumorigenesis and development of GBM. Some of the writers, erasers and even readers of RNA modifications are potential biomarkers for the diagnosis and drug targets for treatment of GBM [30]. Despite the fact that the present study of epi-transcriptome is just a tip of the iceberg, its fast-development will comprehensively expand our sights into cancers.

Nevertheless, most of these studies show that no direct connections of their pro-oncogenic or anti-oncogenic roles are dependent on their capacities of RNA modifications. That is to say that the functions of these modulators in GBM may be not dependent on its function in RNA modifications. Therefore, the RNA modifications and their detailed targets regulated by RMPs should be comprehensively explored in the future. To explore these, much fundamental work should be done before a better understanding of RNA modifications in cancers.

The first task is to identify the physiological and biological function of more than 170 kinds of RNA modifications and their RMPs. However, it is a very challenging and difficult work to decode the complex layer of the epitranscriptome. Although some modifications such as m^6^A can be detected by fast-developing antibody-based NGS through a mechanism of capturing and sequencing of immunoprecipitated RNA targets [35,174,175], however, this technology has some limitations such as low detection accurancy for low-abundance modifications, false-positive mapping results, not-specific capturing antibody, RNA samples contaminations, and Dimroth rearrangement that converse m^1^A to m^6^A [176]. Therefore, it is urgent to develop a novel accurate detecting system that are not dependent on immunoprecipitation.

After solving these problems, exploring the connections of RNA modifications with GBM pathogenesis, prognosis and diagnosis, digging the mechanisms underlying their functions, designing of specific small molecules target RNA modifications, and detecting these targeted drugs penetrating the blood-brain barrier for GBM treatment can be possible. The results of basic research about RNA modifications in GBM will also soon be tested in clinical trials and be applied in the clinic.

## Figures and Tables

**Table 1:** Functions of RNA-modifying proteins (RMPs) in GBM.

Gene Symbol	Gene Description	Role in RNA Modification	Expression	Biological Function	Mechanism	Reference
METTL3	Methyltransferase-like 3	m^6^A writer	Elevated or decreased (controversial)	Maintains or prevents stemness of GSSs; transforms or reverse malignancy; enhances radiosensitivity; reduces migration and induces apoptosis (controversial)	Installs m^6^A marks in GSCs-specifically-expressed genes, such as SOX2; modulates ADAR1 and APOBEC3A to induce A-to I and C-to-U editing; upregulates BCL-X and NCOR2 to affect the levels of SRSFs; prevents YTHDC1-dependent NMD; introduces m^6^A modifications in critical factors, such as ADAM19; downregulates HSP70 expression	[56,57,58,59,60]
METTL14	Methyltransferase-like 14	m^6^A writer	Elevated or Decreased (controversial)	Prevents growth, self-renewal, and tumorigenesis of GSCs	Introduces m^6^A modifications in critical factors, such as ADAM19	[58,59]
WTAP	Wilms’ tumor 1-associated protein	m^6^A writer	Elevated	Stimulates tumorigecity, migration and invasion and inhibits apoptosis	Increases EGFR expression;	[58,61,62,63]
VIRMA/KIAA1429	Virilizer like m^6^A methyltransferase associated protein	m^6^A writer	Decreased	-	-	[58]
ZC3H13/KIAA0853/Flacc	Zinc finger CCCH domain-containing protein 13	m^6^A writer	Decreased	Inhibits malignant tansformation and reduces chemosensitivity	-	[58]
RBM15	RNA binding motif protein 15	m^6^A writer	Elevated	-	-	[58]
RBM15B	RNA binding motif protein 15B	m^6^A writer	Elevated	-	-	[58]
FTO	Fat mass and obesity-associated protein	m^6^A and m^6^A_m_ eraser	Decreased or elevated (controversial)	Promotes self-renewal ability and tumorigenecity of GSCs	Maintains the stability of c-Myc and CEBPA transcripts	[58,59,64]
ALKBH5	α-Ketoglutarate-dependent dioxygenase alkB homolog 5	m^6^A eraser	Elevated	Inhibits cell proliferation of GSCs	Demethylates FOXM1 nascent transcripts to enhance its expression by the aid of lncRNA FOXM1-AS	[58,65]
YTHDF1	YTH N6-methyladenosine RNA binding protein 1	m^6^A reader	Elevated	-	-	[58]
YTHDF2	YTH N6-methyladenosine RNA binding protein 2	m^6^A reader	Elevated	-	-	[58]
YTHDF3	YTH N6-methyladenosine RNA binding protein 3	m^6^A reader	Elevated	-	-	[58]
YTHDC1	YTH domain-containing 1	m^6^A reader	-	Maintains stemness of GSCs	-	[57]
YTHDC2	YTH domain-containing 2	m^6^A reader	Elevated in LGGs; decreased in GBM	-	-	[58]
IGF2BP1/IMP1	Insulin like growth factor 2 mRNA binding protein 1	m^6^A reader	Elevated	Promotes cell proliferation and invasion	Stabilizes the mRNA transcripts of its target genes, including c-Myc, MKI67, PTEN and CD44	[66,67].
IGF2BP2/IMP2	Insulin like growth factor 2 mRNA binding protein 2	m^6^A reader	Elevated	Promotes cell proliferation, migration, invasion and EMT; sensitizes GBM to TMZ treatment; maintains stemness of GSCs; promotes OXPHOS; promotes hypoxia-dependent molecular reprogramming	Activates IGF2-mediated PI3K/AKT signaling; binds to let-7 MREs and prevents LIN28-independent let-7 target gene silencing, including HMGA1, HMGA2, CCND2 and RRM2; binds to several mRNAs such as COX7B, NDUS7, NDUF3 and NDUFS3; interacts with HIF1A-AS2 to maintain HMGA1 expression	[68,69,70,71]
IGF2BP3/IMP3	Insulin like growth factor 2 mRNA binding protein 3	m^6^A reader	Elevated	Promotes cell proliferation, anchorage-independent growth, invasion, EMT and chemoresistance	Activates PI3K/MAPK via promoting IGF2 translation; enhances translation of p65 (RELA)	
eIF3b	Eukaryotic translation initiation factor 3 subunit b	m^6^A reader	Dcreased	Inhibits cell proliferation and induces G0/G1 cell cycle arrest and apoptosis	-	[72]
eIF3h	Eukaryotic translation initiation factor 3 subunit h	m^6^A reader	Dublicated	-	-	[73]
hnRNPA2B1	Heterogeneous nuclear ribonucleoprotein A2/B1	m^6^A reader	Elevated	Promotes GBM cell viability, adhesion, migration, invasion, chemoresistance to TMZ and tumorigenecity as well as protects cells from apoptosis and reactive oxygen species (ROS) generation	Interacts with SOX2; downregulates tumor suppressors such as c-FLIP, BIN1 and WWOX, and upregulates phospho-STAT3, MMP-2, and the proto-oncogene RON	[58,74,75]
hnRNPC	Heterogeneous nuclear ribonucleoprotein C	m^6^A reader	Elevated in LGGs but decreased in GBM	Promotes migratory and invasive activities, increases cell proliferation and protects GBM cells from etoposide-induced apoptosis	Interacts with SOX2; binds directly to pri-mir-21 and promotes the processing of miR-21 that targets PDCD4, supporting Akt and p70S6K activation,	[58,76]
METTL4	Methyltransferase-like 4	m^6^A_m_ writer	Missensely mutated	-	-	[77]
DCP2	Decapping mRNA 2	m^6^A_m_ reader		Participates in radiosensitivity and DNA damage response	-	[78]
TRMT6/TRM6	tRNA methyltransferase 6	m^1^A writer	Elevated	-	-	[79]
TRMT61A/TRM61A	tRNA methyltransferase 61A	m^1^A writer	Elevated	-	-	[79]
ALKBH1	α-Ketoglutarate-dependent dioxygenase alkB homolog 1	m^1^A eraser	Elevated	Promotes cell proliferation and tumorigenecity	Induces DNA demethylation on hypoxia response genes,	[80]
NSUN5	NOP2/Sun RNA methyltransferase 5	m^5^C writer	Decreased	Leads to an adaptive translational program for survival under cellular stress and renders these gliomas sensitive to bioactivatable substrates of the stress-related enzyme NQO1	Drives an overall depletion of protein synthesis	[81,82]
LYREF	Aly/REF export factor	m^5^C reader	Elevated	-	-	[83,84]
TET1	Tet methylcytosine dioxygenase 1	hm^5^C writer	Elevated	-	-	[85]
TET2	Tet methylcytosine dioxygenase 2	hm^5^C writer	Decreased	-	-	[86]
TET3	Tet methylcytosine dioxygenase 3	hm^5^C writer	Decreased	-	-	[87]
ADAR1/ADAR	Adenosine deaminase RNA specific 1	I writer	Decreased	Inhibits cell proliferation	Downregulates A-to-I RNA editing	[88]
ADAR2/ADARB1	Adenosine deaminase RNA specific 2	I writer	Decreased	Inhibits cell proliferation	Downregulates A-to-I RNA editing in the pre-mRNA of a phosphatase CDC14B, thereby downregulates Skp2/p21/p27 proteins	[88,89,90]
ADAR3/ADARB2	Adenosine deaminase RNA specific 3	I writer	Decreased	-	-	[88]
DKC1	Dyskerin pseudouridine synthase 1	ψ writer	Elevated	Promotes cell growth by stimulating cell cycle progression and activates migration	Upregulates N-cadherin, HIF1A, and MMP-2 expression	[91]

*Modification abbreviations*: A-to-I, adenosine-to-inosine; C-to-U, cytidine to uridine, m^1^A, N^1^-methyladenosine; m^5^C, 5-methylcytosine; m^6^A, N^6^-methyladenosine; m^6^A_m_, N^6^, 2′O-dimethyladenosine; hm^5^C, 5-hydroxymethylcytosine; I, inosine; ψ, pseudouridine. *Others:* ADAM19, a disintegrin and metallopeptidase domain 19; ADAR1,adenosine deaminase RNA specific 1; APOBEC3A, apolipoprotein B mRNA editing enzyme catalytic subunit 3A; BCL-X, Bcl-2-like protein 1, BCL2L1; BIN1, bridging integrator 1; c-FLIP, cellular FLICE (FADD-like IL-1β-converting enzyme)-inhibitory protein; c-Myc, avian myelocytomatosis viral oncogene homolog; CCND2, cyclin D2; CDC14B, cell division cycle 14B; CEBPA, CCAAT enhancer binding protein alpha; COX7B, mitochondrial cytochrome c oxidase subunit 7B; EGFR, epidermal growth factor receptor; EMT, epithelial-to-mesenchymal transition; FOXM1, forkhead box M1; FOXM1-AS, forkhead box M1-antisense RNA; GBM, glioblastoma; GSCs, glioma stem-like cells; HIF1A, hypoxia-inducible factor 1 alpha; HIF1A-AS2, hypoxia-inducible factor 1 alpha-antisense RNA 2; HMGA1/2, high mobility group AT-hook 1/2; HSP70, heat shock protein 70; IGF2, insulin-like growth factor 2; LGG, low-grade glioma; MAPK, mitogen-activated protein kinase; MKI67, marker of proliferation Ki-67; MMP-2, matrix metallopeptidase 2; MRE, miRNA recognition element; NCOR2, nuclear receptor corepressor 2; NDUF3,NADH dehydrogenase [ubiquinone] 1 alpha subcomplex assembly factor 3; NDUFS3, mitochondrial NADH dehydrogenase [ubiquinone] iron-sulfur protein 3; NDUS7, NADH dehydrogenase iron-sulfur protein 7; NMD, nonsense-mediated mRNA decay; NQO1, Quaking gene isoform 6; OXPHOS, oxidative phosphorylation; p70S6K, p70 S6 kinase; PDCD4, programmed cell death 4; PI3K, phosphatidylinositol 3-kinase; PTEN, phosphatase and tensin homolog; RON, macrophage stimulating 1 receptor, MST1R; RRM2, ribonucleotide reductase regulatory subunit M2; Skp2, S-phase kinase associated protein 2; SOX2, SRY-box transcription factor 2; STAT3, signal transducer and activator of transcription 3; TMZ, temozolomide; WWOX, WW domain containing oxidoreductase.
